# Characterization of the Secretome from Spheroids of Adipose-Derived Stem Cells (SASCs) and Its Potential for Tissue Regeneration

**DOI:** 10.3390/biomedicines12081842

**Published:** 2024-08-13

**Authors:** Valentina Urrata, Francesca Toia, Emanuele Cammarata, Mara Franza, Luigi Montesano, Adriana Cordova, Anna Barbara Di Stefano

**Affiliations:** 1 BIOPLAST-Laboratory of Biology and Regenerative Medicine-PLASTic Surgery, Plastic and Reconstructive Surgery Section, Department Precision Medicine in Medical, Surgical and Critical Care, University of Palermo, 90127 Palermo, Italyemanuele.cammarata@unipa.it (E.C.); mara.franza@unipa.it (M.F.); adriana.cordova@unipa.it (A.C.); annabarbara.distefano@unipa.it (A.B.D.S.); 2 Plastic and Reconstructive Surgery Unit, Department of Precision Medicine in Medical, Surgical and Critical Care, University of Palermo, 90127 Palermo, Italy

**Keywords:** spheroids of adipose stem cells, extracellular vesicles, secretome, adipose tissue, stemness and mesenchymal differentiation

## Abstract

Introduction: Spheroids are spherical aggregates of cells that mimic the three-dimensional (3D) architecture of tissues more closely than traditional two dimensional (2D) cultures. Spheroids of adipose stem cells (SASCs) show special features such as high multilineage differentiation potential and immunomodulatory activity. These properties have been attributed to their secreted factors, such as cytokines and growth factors. Moreover, a key role is played by the extracellular vesicles (EVs), which lead a heterogeneous cargo of proteins, mRNAs, and small RNAs that interfere with the pathways of the recipient cells. Purpose: The aim of this work was to characterize the composition of the secretome and exosome from SASCs and evaluate their regenerative potential. Materials and Methods: SASCs were extracted from adipose samples of healthy individuals after signing informed consent. The exosomes were isolated and characterized by Dinamic Light Scattering (DLS), Scanning Electron Microscopy (SEM), and Western blotting analyses. The expression of mRNAs and miRNAs were evaluated through real-time PCR. Lastly, a wound-healing assay was performed to investigate their regenerative potential on different cell cultures. Results: The SASCs’ exosomes showed an up-regulation of NANOG and SOX2 mRNAs, typical of stemness maintenance, as well as miR126 and miR146a, related to angiogenic and osteogenic processes. Moreover, the exosomes showed a regenerative effect. Conclusions: The SASCs’ secretome carried paracrine signals involved in stemness maintenance, pro-angiogenic and pro-osteogenic differentiation, immune system regulation, and regeneration.

## 1. Introduction

Stem cells possess several key characteristics that give them a crucial role in tissue development, repair, and regenerative medicine. They are undifferentiated cells with self-renewal ability [1], potency [2,3], and important immunomodulatory properties [4]. They can make symmetric or asymmetric divisions. In the former, the stem cell divides to generate two cells that remain undifferentiated as stem cells, while in asymmetric division, one cell goes on to proliferate and differentiate, and the other one stays as a stem cell [5].

A population of stem cells called mesenchymal stem cells (MSCs) was initially identified in the bone marrow stroma in the late 1960s [6]. Then, MSCs were found in almost all tissues [7,8]. They are a type of multipotent stem cells with important characteristics and properties that make them promising for regenerative medicine and tissue engineering. They are able to differentiate into several mesenchymal lineages, such as adipocytes, osteoblasts, chondrocytes, myocytes [9,10], and also neuron-like [11]. Moreover, they are responsible for several cell functions, such as pro-angiogenesis [12], immunomodulation [13], anti-inflammation [14], anti-apoptosis [15], neuro-protection, and regulation [16], but several studies have shown that these functions are not mainly exerted by cells but by MSCs-secreted paracrine factors [17,18,19,20,21]. In addition, a population of MSCs called adipose-derived stem cells (ADSCs) was also recently investigated thanks to the ease of surgical accessibility of adipose tissue, which also provides a high yield of ADSCs [22]. ADSCs demonstrated significant multilineage differentiation potential and important immunomodulatory activities [23]. Additionally, while 2D cell cultures have long been employed as in vitro models, the focus has recently shifted towards 3D cell cultures due to their superior ability to replicate the in vivo cell microenvironments [24,25,26,27]. In 2D culture conditions, cells grow as a single cell layer on a flat adherent surface, such as a tissue culture plastic dish. This can alter their properties and behavior compared to in vivo conditions. In fact, 2D ADSCs change their morphology and polarity [28]. They can have limited cell–cell interactions and spatial organization [29] and unlimited access to all nutrients of the medium but also each added molecule due to their arrangement on a monolayer [30], and this could alter their gene and protein expression [31]. To overcome these limitations, cells can be cultured in a 3D environment to form suspended aggregates known as spheroids [32]. In 3D cultures, cells grow in a three-dimensional environment that more closely mimics the in vivo tissue architecture, allowing for more natural cell–cell and cell–matrix interactions [33]. An important limitation of 3D cell culture method is the higher complexity and costs compared to 2D cultures. Several techniques have been applied to obtain spheroids, but currently, there is no standardized one [34,35,36]. A recent study showed that spheroids can be directly obtained from liposuction fat or adipose tissue digestion, seeding ADSCs in ultra-low-adhesion conditions without any additional step, forming the so-called SASCs [37]. They showed the ability to maintain stemness until 28 days, with a higher expression of stemness-associated mRNAs as well as better regenerative abilities compared to 2D-cultured cells [38,39]. Moreover, a study revealed that SASCs cultured in Integra scaffold and implanted in an in vivo T8 laminectomy mice model showed a significant involvement in bone tissue regeneration [38], and a similar effect was also obtained after their implantation in an in vivo calvaria rabbit model in which SASCs also stimulated neo-vessels formation [40]. Cells need to communicate with each other and exchange information not only through direct cell–cell interaction but also through indirect methods such as endocrine, autocrine, and paracrine signaling [41]. Paracrine signaling consists of the releasing of soluble factors (cytokines, growth factors, hormones, and extracellular vesicles) by a cell into the extracellular space, which acts on the neighboring cells by affecting their behavior and function [42,43,44]. Amongst the main roles of this signaling are the regulation of cell growth, differentiation, migration [45], maintenance of tissue homeostasis [46], and tissue repair and regeneration [47].

The soluble factors and extracellular vesicles (EVs) secreted by cells into the culture medium are known as the secretome or conditioned medium [48]. Extracellular vesicles (EVs) are membrane-bound structures released by cells into the extracellular space, and they play crucial roles in intercellular communication and various physiological and pathological processes [49,50,51]. EVs are distinguished into exosomes, microvesicles (MVs), and apoptotic bodies, according to their size and origin. Exosomes are spheroidal-shaped vesicles of 30–150 nm in size. They derive from multivesicular bodies (MVBs) generated by the early endosomes. MVBs are rich in intraluminal vesicles (ILVs), generated by the inward budding of endosomal membranes. MVBs’ fate can be dual: They can fuse with lysosomes that are being degraded, or they can be transported to the plasma membrane, and after fusion with it, they can release their EVs content outside the cells. In this case, released ILVs are called exosomes. The proteins mainly expressed on the exosome surface are CD63, CD9, and CD81 [52]. Microvesicles are 100–1000 nm in size and derive from plasma membrane shedding. They are directly released into the extracellular space, and their surface is characterized by membrane components typical of the cell of origin. They are characterized by an irregular shape [53]. Both exosomes and microvesicles are enriched in small and long non-coding RNAs, mRNAs, lipids, and proteins by conveying specific information to the recipient cells [54,55]. Differences in cell properties might lead to the secretion of different factors with the formation of a specific secretome, but few studies have been performed on this issue [34,36,56,57]. The secretome of 3D SASCs has never been characterized, and neither has the role and efficacy of extracellular vesicles in cell communication and their therapeutic applications. We analyzed the secretome from SASCs [58], and in this work, we characterized the exosomal population from SASCs and then screened it for a set of mRNAs and miRNAs as exosomal internal *cargo*. Finally, we assessed the regenerative potential of the total secretome and, in particular, isolated exosomes through a wound-healing assay on endothelial cells, fibroblasts, and osteoblasts.

## 2. Materials and Methods

### 2.1. Cells Extraction and Culture

Adipose tissue or a liposuction sample from different anatomical areas such as the abdomen, hip, and breast were collected from healthy individuals (7 females and 5 males, mean BMI of 27.8, and mean age of 47.0 years) at the Plastic and Reconstructive Surgery Unit of Palermo. The hospital’s ethical committee approved the study, so informed consent was collected from each patient. Adipose tissue or liposuction fat were enzymatically and mechanically digested. Adipose tissue was digested with collagenase (150 mg/mL; Gibco, Carlsbad, CA, USA) and hyaluronidase (20 mg/mL; Sigma, St. Louis, MO, USA) through mechanical agitation for 1 h at 37 °C, while liposuction samples were digested with collagenase (150 mg/mL; Gibco) through mechanical agitation for 30 min at 37 °C. The samples were then centrifuged at 1200× *g* for 5 min, and the stromal vascular fraction (SVF) was divided into two parts: One-half was seeded with serum-free stem cell-specific medium (SCM) added with basic fibroblast growth factor (bFGF; 10 ng/mL; Sigma) and epidermal growth factor (EGF; 20 ng/mL) and plated in ultra-low-adhesion flasks (Corning, Corning, NY, USA). The other half was seeded in adhesion flasks (Corning) with Dulbecco’s modified Eagle’s medium high glucose (DMEM) (Sigma) complemented with 10% fetal bovine serum (FBS) (EuroClone, Milan, Italy) and were called ADSCs. Both the cell media were replaced twice a week. Endothelial cells, fibroblasts, and osteoblasts were cultured in adhesion flasks (Corning). Endothelial cells and osteoblasts were differentiated from SASCs for 21 days with a specific differentiation medium: endothelial cell growth medium (PromoCell, Heidelberg, Germany) and osteoblast growth medium (PromoCell), as previously demonstrated [38,59]. Normal human dermal fibroblasts (NHDF) were provided by PromoCell and cultured with fibroblast growth medium (PromoCell). Cells were maintained at 37 °C in a 5% CO_2_ humidified incubator.

### 2.2. Secretome Collection and Exosomes Extraction

Cells were cultured for one week, and then, the media were collected every 72 h and centrifuged at 2000× *g* per 30 min to remove cells and debris, according to the Total Exosome Isolation (from cell culture media) protocol (Invitrogen, Vilnius, Lithuania). The cell-free culture medium was transferred into a new tube, and 0.5 volumes of total exosome isolation reagent were added. The solution was mixed, and the samples were incubated at 4 °C overnight. Then, the samples were centrifuged at 10,000× *g* for 1 h at 4 °C. The supernatant was discarded, and the pellet was appropriately resuspended in 200 µL 1× PBS for RNA extraction or in 50 µL of exosome resuspension buffer for protein extraction.

### 2.3. Protein Extraction and Quantification

EVs protein extraction was performed by Total Exosome RNA and Protein Isolation Kit (Invitrogen, Carlsbad, CA, USA). After having resuspended the exosome pellet in 50 µL of exosome resuspension buffer for protein extraction, the total protein amount was analyzed through Qubit™ Protein Assay Kit (ThermoFisher, Eugene, OR, USA), according to the manufacturer protocol, and then read using the Qubit™ 4 Fluorometer instrument.

### 2.4. Exosomes Characterization

Exosomes were characterized by dynamic light scattering (DLS), scanning electron microscopy (SEM), and Western blotting analyses. DLS was performed through the Zetasizer nano ZSP 2 (MALVERN Panalytical Ltd., Malvern, UK) instrument and scanning electron microscopy through FEI—Versa 3D Dual-Beam Microscope. In the DLS technique, a laser strikes the solution, and the intensity of the scattered light as a function of time is measured. Light is scattered due to the Brownian motion of particles that correlates with their hydrodynamic diameter. The smaller the particle, the faster it will diffuse. The bigger the particle, the slower it will diffuse. The DLS instrument will generate a correlation function that is mathematically linked with particle size and its time-dependent light-scattering capacity. SEM uses a focused beam of high-energy electrons to investigate the surface of solid samples. Electrons are generated by an electron source and are accelerated. When they impact against the sample, they are decelerated. The electron–sample interactions produce a variety of signals, such as backscattered electrons, secondary electrons, photons, and visible light and heat. All of them give information about the sample’s external morphology and crystalline structure and orientation at the micro and nano scale.

For Western blotting, after protein extraction and quantification, 40 µg of proteins from SASCs-derived exosomes were complexed with 2× Laemmli sample buffer (BioRad, Hercules, CA, USA) and added with 2-mercaptoethanol (BioRad). The same protein amount from 3D SASCs and 2D ASCs as control was prepared. Samples were loaded in the Mini-PROTEAN^®^ TGX Stain-Free™ Gels (BioRad). The gel was blotted on the Trans-Blot^®^ Turbo™ Transfer Pack (BioRad) through the Trans-Blot Turbo instrument (BioRad) for 7 min. The stain-free technology was used to evaluate the protein separation in the gel and the membrane. This was then incubated with 1× TBS 1% casein blocker (BioRad) for 1 h at RT during shaking and incubated overnight at 4 °C with the primary antibody CD63 (Invitrogen) diluted 1:250. The day after, the membrane was washed three times with tTBS 0.05% and then incubated for 2 h with the goat anti-mouse IgG antibody (HRP) (GeneTex, Inc., Irvine, CA, USA) diluted 1:5000. The membrane was washed with tTBS 0.05% twice and TBS 1× once, and then, the Clarity MaxTM Western ECL substrate (BioRad) was prepared by mixing in a 1:1 ratio the Clarity Western Peroxide Reagent and the Clarity Western Luminol/Enhancer Reagent. The membrane was incubated in the substrate solution for 5 min and then exposed to the ChemiDoc Imaging System (BioRad).

### 2.5. mRNA Extraction from Exosomes

mRNAs from exosomes were extracted by RNeasy Mini Kit (Qiagen, Hilden, Germany). First, 350 µL of buffer RLT previously completed with 2-mercaptoethanol (BioRad), was added to the pellet. The mix was vortexed for 1 min and passed through a 1 mL syringe with a needle. Then, 1 volume of 70% ethanol was added, and the mixture was transferred to an RNeasy Mini Spin column and centrifuged at 12,000 rpm for 15 s. The flowthrough was discarded, and a new centrifuge with the addition of 700 µL of Buffer RW1 onto the RNeasy Mini Spin column was performed for 15 s at 12,000 rpm. The flowthrough was discarded, and two other centrifugations with the addition of 500 µL of buffer RPE were performed at the same velocity of 12,000 rpm but, respectively, for 15 s and 2 min. Then, the RNeasy Mini Spin column was centrifuged at full speed (14,000 rpm) for 1 min to dry the membrane. Finally, 30 µL of RNase-free water was added directly to the column membrane and centrifuged for 1 min at 10,000 rpm. The total mRNA was eluted.

### 2.6. miRNA Extraction from Exosomes

miRNAs from exosomes were extracted by miRNeasy Mini Kit (Qiagen). The exosome pellet was disrupted by adding QIAzol Lysis Reagent and homogenized by vortexing for 1 min and by passing it through a 1 mL syringe with a needle. After incubating the homogenate for 5 min at RT, 140 µL of chloroform was added and shaken for 15 s. The sample was incubated for 3 min at RT and then centrifuged at 12,000× *g* for 15 min at 4 °C. The upper aqueous phase was recovered, and 1.5 volumes of 100% ethanol were added. Next, 700 µL of the sample was transferred onto the RNeasy Mini Spin column and centrifuged at 12,000 rpm for 15 s at RT. The flowthrough was discarded. To wash the column, 700 µL of buffer RWT was added and centrifuged at 12,000 rpm for 15 s. Then, 500 µL buffer RPE was pipetted onto the RNeasy Mini spin column and centrifuged for 15 s at 12,000 rpm to wash the column. The flowthrough was discarded, and the previous step was performed again. A full-speed centrifuge for 1 min was executed to eliminate any possible contaminant inside the RNeasy Mini spin column. Finally, the RNeasy Mini spin column was transferred into a new 1.5 mL collection tube, and 30 µL RNase-free water was pipetted directly onto the membrane. It was centrifuged for 1 min at 12,000 rpm to elute the RNA.

### 2.7. RNA Quantification

RNA was quantified by Qubit™ RNA HS Assay Kit (ThermoFisher) according to the manufacturer protocol and then read using the Qubit™ 4 Fluorometer instrument.

### 2.8. mRNA Reverse Transcription and Real-Time PCR

To perform mRNA reverse transcription, the High-Capacity cDNA Reverse Transcription Kit (ThermoFisher) was used. The sample was mixed as follows: 10× RT buffer 5 µL, 25× dNTP Mix 2 µL, 10× RT random primers 5 µL, H_2_O 10.5 µL, MultiScribe™ Reverse Transcriptase 2.5 µL, and 25 µL of sample. The reverse transcription was performed by the MiniAmp Plus Thermal Cycler (ThermoFisher), and the protocol for cDNA obtaining was the following: 10 min at 25 °C, 2 h at 37 °C, and 5 min at 85 °C, followed by a decrease to 4 °C to remove the sample.

To perform real-time PCR, a master mix was prepared as follows: 10 µL of TaqMan™ Fast Advanced Master Mix (ThermoFisher), 7 µL of H_2_O, 1 µL of primer, and 2 µL of sample for a total of 20 µL per well. The amplification protocol was the following: Step 1 consisted of 2 min at 50 °C and 2 min at 95 °C, and step 2 consisted of 40 cycles, each one comprising 1 s at 95 °C and 20 s at 60 °C. Real-time PCR was executed through the StepOnePlus instrument (ThermoFisher). The evaluated mRNAs were as follows: *Sox2* (Hs01053049_s1), *Nanog* (Hs04399610_g1), *Pou5f1* (Hs00999632_g1), *Prom1* (Hs01009259_m1), *Sox9* (Hs01001343_g1), *Vegfa* (Hs00900054_m1), *Hif1a* (Hs00153153_m1), *Pparg* (Hs01115513_m1), *Runx2* (Hs00231692_m1), *Vegfr2* (Hs00911700_m1), *Igf1* (Hs01547656_m1), *Cd31* (Hs00169777_m1), and *Gapdh* (Hs02758991_g1). Results were standardized to the relative expression of GAPDH.

### 2.9. miRNA Reverse Transcription and Real-Time PCR

To perform miRNA reverse transcription, the TaqMan™ MicroRNA Reverse Transcription Kit (ThermoFisher) was used. Firstly, a primer pool containing 10 primers was generated. The primer pool was a mixture made by 2.5 µL of each primer and 225 µL of H_2_O. Then, the reaction mix was prepared as follows: 6 µL of primer pool, 0.30 µL of dNTP mix w/dTTp, 1.5 µL of 10× RT buffer, 0.19 µL of RNase inhibitor, 3 µL of MultiScribe™ RT enzyme, and 4.04 µL of sample, corresponding to 19 ng of RNA for a total volume of 15 µL for the reverse transcription.

The reverse transcription protocol for miRNAs was the following: 30 min at 16 °C, 30 min at 42 °C and 5 min at 85 °C. Then, the temperature was decreased to 4 °C to allow the removal of the sample. To perform real-time PCR, a master mix was prepared as follows: 5 µL of TaqMan™ Fast Advanced Master Mix (ThermoFisher), 3.84 µL of H_2_O, 0.67 µL of sample, and 0.50 µL of primer for a total of 10 µL per well. The amplification protocol was divided into two steps: 20 s at 95 °C was the first step, and then, 3 s at 95 °C and 30 s at 60 °C, both repeated 40 times, was the second one. Real-time PCR was executed through the StepOnePlus instrument (ThermoFisher).

The evaluated miRNAs were as follows: hsa-miR-191 (TM/RT:002299), mmu-miR-451 (TM/RT:001141), hsa-miR-126 (TM/RT:002228), hsa-miR-100 (TM/RT:000437), hsa-miR-221 (TM/RT:000524), mmu-miR-495 (TM/RT:001663), mmu-miR-140 (TM/RT:001187), hsa-miR-30c (TM/RT: 000419), hsa-miR-143 (TM/RT: 002249), hsa-miR-146a (TM/RT: 000468), hsa-miR-142-3p (TM/RT: 000464), and hsa-miR-182 (TM/RT: 002334). Results were standardized to the relative expression of miR-191.

### 2.10. Wound-Healing Assay

Endothelial cells, fibroblasts, and osteoblasts were seeded in a 24-well plate at the concentration of 15,000 cells/well until they reached confluence. The experimental conditions were the following: control exosomes (specific cell culture medium + PBS), control secretome (specific cell culture medium + SCM), total exosomes (specific cell culture medium + isolated exosomes), and total secretome (specific cell culture medium + total secretome from 3D-cultured SASCs). Image J software 1.8.0 was used to measure the percentage of wounded area.

### 2.11. Statistical Analysis

Data are expressed as mean ± standard deviation of three independent experiments. Statistical significance was calculated using one way analysis of variance (ANOVA), followed by either a Tukey’s or Bonferroni’s multiple comparison post hoc test. Significance levels were analyzed with GraphPad Prism 8 statistical software and indicated as *p*-values (* *p* < 0.05, ** *p* < 0.01, and *** *p* < 0.001).

## 3. Results

### 3.1. Exosomes Characterization

**Figure 1:**
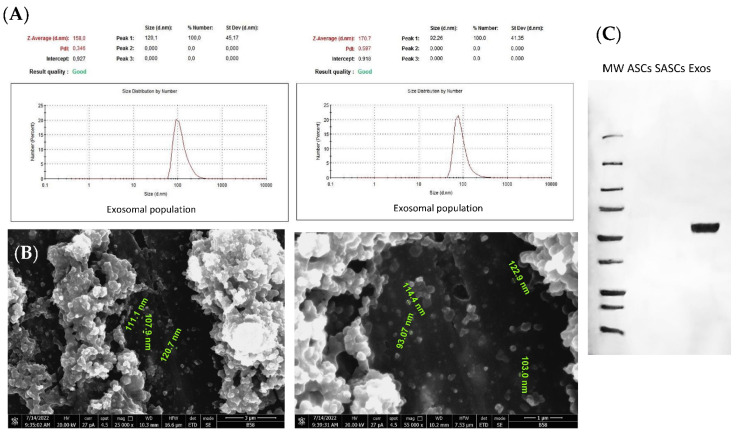
Exosomes characterization. (**A**) DLS analysis for physical characterization, (**B**) SEM analysis for exosomal size and shape characterization, and (**C**) CD63, typical exosomal expression marker, by Western blotting analysis.

DLS analysis was performed to assess the uniformity of the exosomal population extracted from the secretome of SASCs. The results showed that all the analyzed exosomal populations were consistent in size and ranged between 80 and 120 nm, in agreement with the known exosomal dimensions amongst 30 and 150 nm [60] (Figure 1A). SEM analysis was employed to investigate the exosomes’ morphology and dimensions, demonstrating their characteristic round shape and a size between 90 and 120 nm that is typical of exosomes, which corroborates the DLS results (Figure 1B). The protein expression analysis confirmed the presence of a rich exosomal population derived from SASCs, marked by CD63 positivity. The proteins from cell lysates of 2D ASCs and 3D SASCs were used as controls (Figure 1C).

### 3.2. mRNA Analysis

**Figure 2:**
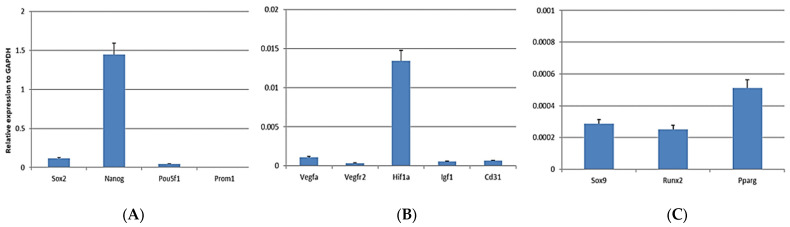
Exosomal internal *cargo*: analysis of 12 mRNAs. (**A**) Stemness-related mRNAs (*Sox2*, *Nanog*, *Pou5f1* and *Prom1*); (**B**) angiogenesis-related mRNAs (*Vegfa*, *Vegfr2*, *Hif1a*, *Igf1* and *Cd31*); (**C**) mesenchymal differentiation-related mRNAs (*Sox9*, *Runx2* and *Pparg*).

The gene expression analysis was performed to screen SASCs derived exosomes for a pool of mRNAs as their internal *cargo*. The results indicated that exosomes significantly expressed stemness-related mRNAs compared to the ones related to angiogenesis or mesenchymal differentiation. Specifically, *Nanog* was the most highly expressed mRNA amongst the stemness-related ones, followed by *Sox2* and *Pou5f1* (Figure 2A). Among the angiogenesis-related mRNAs, *Hif1a* was the most prominently expressed compared to *Vegfa*, *Vegfr2*, *Igf1* and *Cd31* which were equally poorly expressed (Figure 2B). Conversely, the mRNAs related to mesenchymal differentiation, representative of chondrocytic (*Sox9*), osteoblastic (*Runx2*), and adipocytic (*Pparg*) lineages, were poorly expressed at comparable levels (Figure 2C).

### 3.3. miRNA Analysis

**Figure 3:**
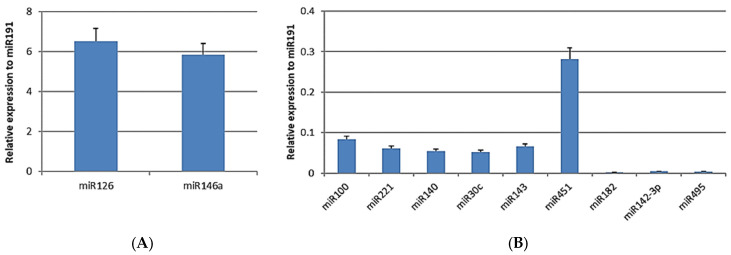
Exosomal internal *cargo*: analysis of 11 miRNAs. (**A**) More-expressed miRNAs (miR126 and miR146a); (**B**) less-expressed miRNAs (miR100, miR221, miR140, miR30c, miR143, miR451, miR182, miR142-3p, and miR495).

A set of 11 microRNAs (miRNAs), small endogenous non-coding RNAs of approximately 22 nt in length, was screened as a part of the exosomal internal *cargo* [61,62]. We analyzed the representative miRNAs related to angiogenesis (miR126), stemness (miR142-3p), osteoblastic (miR100 and miR221), chondrocytic (miR140 and miR495) and adipocytic (miR30c and miR143) differentiation, and immunomodulation (miR146a, miR451, and miR182). Among these, miR126 and miR146a exhibited the highest levels of expression (Figure 3A), followed by miR451. SASCs-derived exosomes expressed lower levels of miR100, miR143, miR221, miR140, and miR30c and even loss of miR182, miR142-3p, and miR495 (Figure 3B).

### 3.4. Wound-Healing Assay

**Figure 4:**
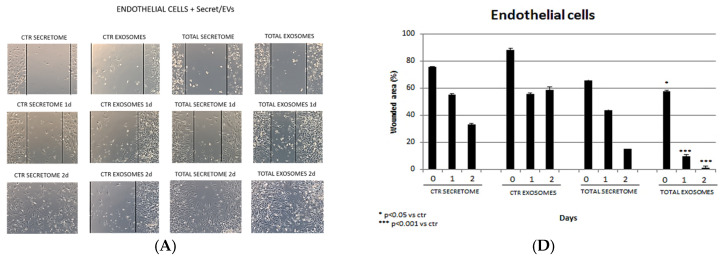

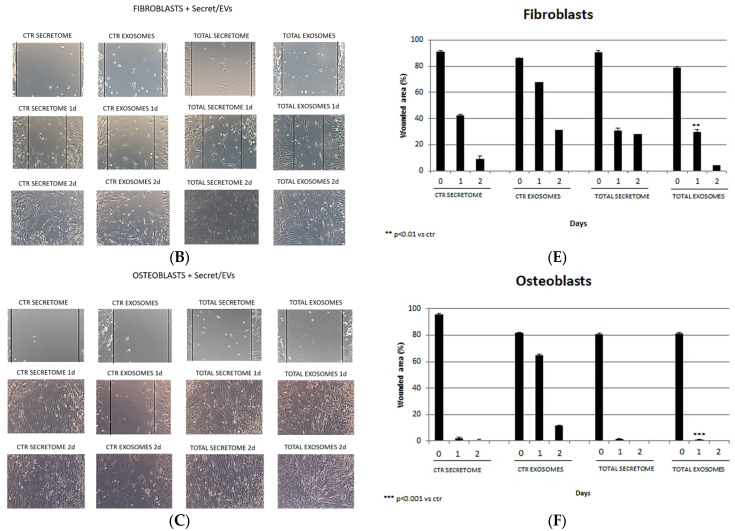
Wound-healing assay on (**A**) endothelial cells, (**B**) fibroblasts, and (**C**) osteoblasts (20× magnification). In the first two columns, the controls (secretome and exosomes) are shown. In the second two columns, total secretome and isolated exosomes added to the cell specific culture media are shown. Percentage of the wounded area on (**D**) endothelial cells, (**E**) fibroblasts, and (**F**) osteoblasts at 0, 1, and 2 days.

A wound-healing assay was performed to investigate if the SASCs-derived secretome or exosomes exerted a regenerative potential on several cell cultures. The endothelial cells treated with the secretome did not exhibit a significant wounded area closure after 1 day of treatment compared to the control. However, treatment with the total exosomes led to a notable reduction in the wounded area (80%) after 1 day, with complete wound closure observed after 2 days (Figure 4A,D). Similarly, fibroblasts showed no substantial wound closure after 1 day of treatment with the secretome compared to the control, while a significant reduction in the wounded area (60%) was achieved with the total exosomes, leading to near-total closure by day 2 (Figure 4B,E), demonstrating an active proliferation of cells. Osteoblasts treated with the secretome showed a total closure of the wound due to rapid cell proliferation compared to the control. In contrast, total exosomes resulted in complete wound closure after just 1 day of treatment (Figure 4C,F).

## 4. Discussion

It has been already demonstrated that cells cultured in 3D conditions exhibit superior properties compared to those grown in 2D adhesion conditions. In fact, in 3D cultures, cells can better mimic their native conditions, preserving their surface characteristics and maintaining their gene and protein expression profiles due to the loss of interactions with the surface of the culture plate [31].

Several techniques have been developed to obtain spheroids even if, to date, there is no standardized protocol [34,35,36]. In this study, spheroids termed SASCs were directly obtained from liposuction fat or adipose tissue digestion by seeding ADSCs under ultra-low-adhesion conditions without additional steps. SASCs were already characterized as showing the ability to maintain stemness for up to 28 days, with a higher expression of stemness-associated mRNAs as well as enhanced regenerative abilities compared to 2D-cultured cells [38,39]. Cells need to exchange information each other, and to do so, they communicate not only through direct interactions but also through endocrine, autocrine, and paracrine signaling [41]. Paracrine signaling involves the secretion of soluble factors and extracellular vesicles (EVs) into the culture medium, known as the conditioned medium or secretome. The secretome offers a higher safety profile compared to cell engrafting due to its lower risk of neoplastic transformation [63] and easier storage through the use of natural and non-toxic agents such as trehalose, a natural disaccharide found in many foods [64]. In recent years, there has been a growing focus on extracellular vesicles (EVs) such as exosomes due to their potential in clinical applications as diagnostic biomarkers and therapeutic carriers [40]. Exosomes are particularly advantageous because of their biocompatibility, which reduces immunogenicity, and their bi-layered lipid structure, which also protects their *cargo* from degradation. In addition, their small size and membrane composition allow them to cross major biological membranes, including the blood–brain barrier [52].

A recent study conducted by our group analyzed the composition of the secretome from SASCs by screening it for a panel of soluble factors [58]. We found that SASCs secreted significant concentrations of growth factors related to stemness maintenance and angiogenesis, such as PIGF-1 (placental-derived growth factor), HGF (hepatocyte growth factor), and FGF-2 (fibroblast growth factor 2) and several interleukins (ILs) related to the immune system modulation, such as IL-5, IL-1Ra, IL-8, IL-2, IL-7, IL-23, IL-15, IL-13, IL-10, and the chemokine CCL-4. In addition, typical endothelial factors such as VEGFR2, CD31, CD62E, and ICAM-1 were overexpressed. The scientific literature has shown that PIGF-1 plays a role in inducing angiogenesis in vivo and promoting the proliferation and migration of endothelial cells in vitro [65]. Other studies have demonstrated that HGF is crucial for maintaining the stemness of hBM-MSCs [66] as well as for inducing angiogenesis [67]. Similarly, FGF-2 has been shown to play a role in maintaining the stemness of BM-MSCs [68] and preventing cellular senescence [69]. Our previous data indicate that SASCs are able to communicate with each other through paracrine signaling, likely promoting stemness through the secretion of typical growth factors involved in this process. In addition, amongst the most-expressed interleukins, they can be conventionally grouped as pro- and anti-inflammatory or adaptive immunity, but all of them are involved in immune system modulation. A review demonstrated that interleukins can exert a pro- or anti-inflammatory effect depending on their concentration as well as the nature of the target cell and the activating signal or its timing [70]. Thus, the secretion of all these immunomodulatory molecules makes it possible to ascribe to SASCs’ secreted factors an important role in balancing immune system activation and deactivation, also considering the hypothesis that varying cell concentrations or cultivation times could alter the concentration of released interleukins, influencing their role in the immune response. The expression of typical endothelial analytes as soluble factors secreted by SASCs could suggest an enrichment of extracellular vesicles (EVs) in the conditioned medium and their expression of typical endothelial markers. This could be due to the high angiogenic potential of SASCs [59] that might lead cells to generate EVs that carry typical angiogenic molecules. DLS, SEM, and Western blotting analyses confirmed the presence of an exosomal population within the 30–150 nm size range, exhibiting a round shape and enrichment with CD63 as membrane marker. A pool of mRNAs was then analyzed as exosomal internal *cargo*, and we found a high expression of *Nanog*, followed by *Sox2* and *Pou5f1*, which are involved in stemness maintenance. *Nanog* is a gene located on chromosome 12 and codifies for a transcription factor crucial for maintaining stemness in both embryonic pluripotent cells and adult MSCs [71,72]. Moreover, it regulates the expression of several factors involved in the maintenance of the immunomodulatory functions of MSCs [73]. Similarly, *Sox2* encodes for a transcription factor with a key role in MSC stemness maintenance and proliferation [74]. In addition, it is responsible for cell growth and differentiation towards adipogenic, osteogenic, and chondrogenic lineages in hMSCs [75]. *Pou5f1* is usually expressed by embryonic stem cells and codify for a key transcription factor involved in the maintenance of self-renewal and undifferentiated state [76]. These results align with the stemness condition of SASCs, suggesting that they may likely regulate their self-renewal and maintenance of their undifferentiated state through paracrine signaling. For regenerative applications, using their exosomes could be beneficial, as they carry stemness messages that could be directly injected in the damaged site and diffuse into the neighboring tissues way to promote the proliferation of resident stem cells. Furthermore, it could be advantageous to employ simultaneous use of both *Nanog* and *Sox2* mRNAs to modulate the immune system. The analysis of a pool of 11 miRNAs as exosomal internal *cargo* revealed high levels of miR126 and miR146a, followed by lower levels of miR451, miR100, miR143, miR221, miR140, and miR30c. Several studies have shown that miR126 supports endothelial cell angiogenesis [77] by promoting endothelial differentiation of BM-MSCs, increasing CD31, eNOS, and VE-cadherin levels or directly inhibiting PIK3R2 (phosphoinositol-3 kinase regulatory subunit 2) and SPRED1 (Sprouty-related protein), two negative regulators of the VEGF signaling pathway [78]. Additionally, miR-126 and miR-146a together have been shown to induce cardiac regeneration, with miR126 aiding in cell migration and angiogenesis and miR146a providing anti-inflammatory activity. In the study, exosomes from ADSCs loaded with these miRNAs were encapsulated in injectable Alg hydrogel and injected in myocardial infarction animal models. The authors found that the miR-126 and miR-146a combination led to the improvement of HUVECs migration and proliferation as well as angiogenesis, promoting an overexpression of Connexin 43 (CX43) and VEGFR2 together with PI3K-AKT pathway activation and a decrease in inflammation mediated by miR146a via inhibiting TRAF-6 and IRAK-1 [79]. In addition, the overexpression of miR146a in BM-MSCs transfected with miR146 enhanced cell proliferation, migration, and osteogenic differentiation in canine right-mandibular distraction osteogenesis (DO) models [80]. Moreover, several studies proved that miR146a was also a negative regulator of inflammation [81,82,83]. These results suggest that the balance between miR146a and miR126 inside exosomes could probably give an equal contribution to the proliferation, migration, and differentiation of endothelial cells and osteoblasts, and it also could modulate immune system responses if the exosomes were used in in vivo applications. Amongst the less-expressed miRNAs, miR451 was studied in the tumor field, acting as a suppressor of osteosarcoma and hepatocellular carcinoma growth and angiogenesis [84,85]. miR100 was negatively related to angiogenesis in endothelial and vascular smooth muscle cells by negatively regulating VEGF [86] and mTOR signaling, which is responsible for sprouting phenomena, tube formation, and proliferation of endothelial cells [87]. In SASCs, the expression of miR100 increases during late osteogenic differentiation, while miR221 is down-regulated [39]. miR143 has been positively related to osteoblasts differentiation and pro-angiogenic activity [88], while miR140 has been only studied in chondrocytes, where it is expressed during embryonic bone development [89], playing a crucial role in cartilage matrix stability and chondrocyte senescence inhibition [90]. miR30c targets IL-6, a typically pro-inflammatory cytokine [91]. Since they have not yet been studied, all these findings open new perspectives for the more in-depth study of the effects of each of these miRNAs on angiogenesis and osteogenesis in the regenerative field. Moreover, we could speculate that the high concentration of miR-126 with pro-angiogenic functions within SASCs-derived exosomes could probably induce some cells to show their angiogenic differentiation potential, which is already widely demonstrated [59]. This could explain why SASCs could generate exosomes with typical endothelial membrane markers. Finally, the wound-healing assay on endothelial cells, fibroblasts, and osteoblasts demonstrated that in all three cell lineages, exosomes significantly reduced the wounded area within one day, leading to nearly complete wound closure after two days.

Although the exosomes can currently be considered an ideal candidate for clinical applications (diagnosis, progression, and therapy) as a new therapeutic strategy with no immunogenicity, toxicity, and risk while also offering higher stability, maintenance, and integration compared to origin cells, several limitations are related to their study and use [92]. The lack of standardized techniques for their isolation and purification and established characterization methods requires further studies in this regard [93]. Above all, the main limitation is the need for large quantities of starting samples, in our case adipose tissue, from which to isolate cells to obtain the amount of exosomes sufficient for the experiments. To resolve this but also to minimize heterogeneity and variability in the results, we used samples from different patients, in line with our previous studies in which we demonstrated the homogeneity of isolated SASCs. Several strategies could be developed to use exosomes as a carrier by using biomolecules as cell stimulators to increase the exosomes yield [94]. In addition, diseased or aged tissues should be some important parameters to consider because they could alter the *cargo* of the isolated exosomes, which would impact the results of the study. In addition, the microenvironment created by the target cells could also play a significant role in the reparative response of wounded tissue, influencing the amounts of inflammatory mediators produced and thus the regenerative capacity of the tissue itself. However, the use of functionalized EVs might be considered in clinical settings to enhance the regenerative effect of cells rather than the reparative one. Indeed, in a dynamic in vivo environment, functionalized exosomes could be used for specific targeting of stem cells adjacent to damaged tissues to induce their proliferation and differentiation and to actively stimulate a regenerative response that creates a microenvironment maintained by signaling cues.

Despite these limitations, our findings suggest that SASCs-derived exosomes hold promise for regenerative purposes, particularly in tissue regeneration and vascularization applications. Further studies are also needed to evaluate the in vivo efficacy and safety of SASCs-derived exosomes in regenerative medicine.

Moreover, they also showed a positive regenerative effect on fibroblasts. This could allow the formation of new connective tissue and new extracellular matrix in in vivo treatments, enabling the maintenance of the homeostasis in regenerated tissues.

## 5. Conclusions

This work aimed to provide an initial characterization of the secretome from SASCs. At first, analysis of the exosomal *cargo* revealed a high expression of *Nanog* mRNA, followed by *Sox2*, both of which are related to stemness. Additionally, miR126, associated with angiogenesis, and miR146a, associated with osteogenesis, were overexpressed. Their balanced expression levels might lead to a combined pro-osteogenic and pro-angiogenic effect on the recipient cells. Finally, the wound-healing assay demonstrated the pro-regenerative effects of SASCs-derived exosomes on endothelial cells, fibroblasts, and osteoblasts within just 1 day of treatment. These findings suggest that spheroids of adipose-derived stem cells secrete factors involved in stemness maintenance, immunomodulation, and pro-differentiation signals towards endothelial or osteoblastic lineages, making them promising candidates for future in vivo regenerative studies.

## Data Availability

The original contributions presented in the study are included in the article, further inquiries can be directed to the corresponding author.
